# Physical Activity and Self-Perception of Mental and Physical Quality of Life during Pregnancy: A Systematic Review and Meta-Analysis

**DOI:** 10.3390/jcm12175549

**Published:** 2023-08-25

**Authors:** Miguel Sánchez-Polán, Kristi Adamo, Cristina Silva-Jose, Dingfeng Zhang, Ignacio Refoyo, Rubén Barakat

**Affiliations:** 1 AFIPE Research Group, Faculty of Physical Activity and Sport Sciences-INEF, Universidad Politécnica de Madrid, 28040 Madrid, Spain; 2 School of Human Kinetics, Faculty of Health Science, University of Ottawa, Ottawa, ON K1N 6N5, Canada; 3 Sports Department, Faculty of Physical Activity and Sports Sciences-INEF, Universidad Politécnica de Madrid, 28040 Madrid, Spain

**Keywords:** self-perception of health status, quality of life, mental/psychological, physical, pregnancy, physical activity

## Abstract

Self-perception of health status (quality of life) is considered one of the best indicators of health and well-being. However, during pregnancy, it could be impacted not only by physiological and anatomical changes, but by poor lifestyle habits like high sedentary behaviour or bad nutrition. This study assesses the effects of physical activity RCT interventions during pregnancy on both mental and physical components of quality of life. A systematic review and two meta-analyses were performed (PROSPERO registration number: CRD42022370467). Of the 207 articles captured both in English and Spanish, seven articles were deemed eligible for inclusion. The two analyses performed found that physically active pregnant women had better scores of mental self-perception of quality of life (z = 2.08, *p* = 0.04; SMD = 0.34, 95% CI = 0.02, 0.67, I^2^ = 76%, P_heterogeneity_ = 0.0004) and in physical self-perceived health status (z = 2.19, *p* = 0.03; SMD = 0.33, 95% CI = 0.03, 0.63, I^2^ = 71%, P_heterogeneity_ = 0.002) compared to control group pregnant women. Physical activity interventions could potentially increase mental and physical self-perception of quality of life during pregnancy.

## 1. Introduction

Pregnancy, arguably one of the most important periods in human life, could be associated with a plethora of physiological and anatomical changes that also could severely alter one’s self-perception and, subsequently, mental health. Reports indicate an upward trajectory in emotional pathologies, with a prevalence of prenatal depression, anxiety, or stress [1,2] being higher now than before the COVID-19 pandemic. With a concerning increase in these pathologies, logic dictates that the perception that pregnant women have about themselves, their health status, and physical conditioning could also be altered, affecting quality of life (QoL). The World Health Organization defines QoL as “an individual’s perception of their position in life in the context of the culture and value systems in which they live and in relation to their goals, expectations, standards and concerns” [3]. We know QoL changes across trimesters of pregnancy, with women achieving the best perception about themselves and their health and mental status in the second trimester, and the lowest in the late third trimester [4], coinciding with the moment of greatest emotional vulnerability experienced during pregnancy [5]. As such, it is important to examine factors that impact QoL in pregnancy, such as primiparity, maternal age, early gestational age, and social or economic problems, among others [6].

QoL not only depends on the time course of pregnancy but can be detrimentally affected by factors such as nausea, sleep difficulties, environment factors (i.e., tobacco exposure/intake) [7], financial challenges, lumbopelvic pain [8], or domestic violence [6]. Additionally, changes in body mass index, weight gain (specifically, excessive gains), or other complications can negatively influence one’s self-perception of health status [9]. Evidence suggests that those experiencing suboptimal QoL in pregnancy are more likely to experience mental health challenges (e.g., depression) [10] and restrict their physical activity (PA) [11]. Unfortunately, mental health pathologies and sedentarism can increase the likelihood of metabolic, cardiovascular, physiological, or even psychological comorbidities [12].

Given the relationship between mental health status and QoL, strategies to effectively attenuate these pathologies during pregnancy should beneficially impact QoL. In this context, as physical activity continues to be integrated into the daily routines of diverse populations, including pregnant individuals, acquiring substantiated evidence regarding the ramifications of prenatal physical activity on women’s quality of life stands as a matter of significant scientific intrigue. However, exploration into this realm remains limited in the current literature. A comprehensive examination of the impact of physical activity during pregnancy reveals consistently favorable outcomes, a notion corroborated by recent investigations [13]. It has been demonstrated that regular physical activity during pregnancy can assist with managing weight gain [14], reducing the intake of harmful substances [15], and decreasing pregnancy complications (e.g., gestational diabetes mellitus, hypertension disorders of pregnancy, and instrumental deliveries) [14,16], but also could help dampen the prevalence of psychological–emotional pathologies [17,18]. This systematic review and meta-analysis aimed to synthetize the scientific literature and assess the effects of physical activity interventions on both physical and mental dimensions of QoL during pregnancy compared with non-physically active women.

## 2. Materials and Methods

This systematic review was performed under the Preferred Reporting Items for Systematic Reviews and Meta-Analyses guidelines (PRISMA) and registered in the International Prospective Register of Systematic reviews (PROSPERO) with the following registration number: CRD42022370467.

### 2.1. Population

Pregnant women without obstetrical contraindication to exercise (as outlined in various published international clinical guidelines of physical activity during pregnancy) participating in a physical activity intervention during pregnancy were the target population [19,20].

### 2.2. Intervention

Any type of measurable physical activity intervention during pregnancy (excluding physical activity advice only without the delivery of an exercise program) which included more than one study group to compare intervention results was eligible. Where applicable/available, studies with co-intervention (e.g., nutrition advising or intervention, or other type of intervention) were retrieved for descriptive purposes, but not analyzed. 

### 2.3. Comparison

Pregnant women who were not engaged in a physical activity intervention (commonly assigned to control or usual care group, receiving usual care follow-up but not involving exercise during pregnancy) were compared with intervention group women.

### 2.4. Outcomes

Eligible study outcomes were physical and mental self-perception of health status. Although there is no scientific consensus on the use of a specific questionnaire to assess the quality of life of pregnant women, we decided to analyze the most commonly used validated tools, which are: Short Form 8 (SF-8), Short Form 12 (SF-12), and Short Form 36 (SF-36) Health Surveys and the World Health Organization Quality of Life questionnaire in its brief version (WHOQOL-BREF), each with 0–100 as a minimum and maximum score. 

### 2.5. Study Design

To perform this systematic review, only studies with a randomized control trial design, involving a quantifiable physical activity intervention and with previously described outcomes of interest included, were eligible. 

### 2.6. Search Strategy

A comprehensive literature search was performed using the following databases: EBSCO (including Academic Search Premier, Education Resources Information Center, MEDLINE, SPORTDiscus, and OpenDissertations databases), ClinicalTrials.gov, Web of Science, Scopus, the Cochrane Database of Systematic Reviews, and the Physiotherapy Evidence Database (PEDro). Articles published between 2010 and 2022 written in English and Spanish were retrieved. The search began in October 2022 and ended in November 2022. Search terms were:

- English: Health status perception or quality of life or health status AND pregnancy or pregnant or prenatal or antenatal or perinatal or maternal AND exercise or physical activity or fitness or sport aerobic training or strength training or cardiovascular training AND randomized controlled trials or rct or randomised control trials or randomized AND intervention.
- Spanish: Percepción del estado de salud o calidad de vida o estado de salud Y embarazo o embarazada o prenatal o antenatal o perinatal o materno Y ejercicio o actividad física o fitness o deporte o entrenamiento aeróbico o entrenamiento de fuerza o entrenamiento cardiovascular Y ensayo clínico aleatorizado o eca o ensayo controlado aleatorizado o aleatorizado Y intervención.

Initially, titles and abstracts were screened by two reviewers and full articles were retrieved if they were deemed to meet the criteria. Full-text articles were reviewed, and if the article did not meet the inclusion criteria, it was reviewed by others to ensure exclusion was warranted. Data were independently extracted from articles meeting our inclusion criteria by one researcher and later reviewed by two different researchers. In cases where the mean or standard deviation of the target outcomes was not reported or data were not available for extraction, the authors were contacted; if no answer was received, the articles were discarded. 

Table 1 displays the author(s), year of publication, country, sample size in each study group, intervention characteristics (frequency of weekly sessions; intensity; program duration; type of physical activity intervention; supervision of the program; duration of individual sessions; and, if available, adherence), main and secondary outcomes analyzed within the studies, and, if possible, co-intervention.

### 2.7. Quality-of-Evidence and Risk-of-Bias Assessments

The Grading of Recommendations Assessment, Development and Evaluation (GRADE) framework using its GRADEpro Guideline Development Tool was used to assess the quality of evidence [21,22]. Risk of bias in RCTs was assessed following the *Cochrane Handbook* [23]. Selected studies were examined for potential bias related to selection, performance, attrition, detection, and reporting. The risk of bias was categorized as low, high, or unclear risk for each source in all the articles. One researcher performed these assessments followed by two different assessors for confirmation and consensus. 

**Table 1:** Characteristics of analyzed articles.

Ref	Country	N	IG	CG	Intervention							Main Variables	Secondary Variables
					Freq	Intens	Time	Type	Superv	Durat	Adh		
Arizabaleta et al., 2010 [24]	Colombia	50	24	26	3	Mod	12 w	Aerobic, stretching, and relaxation exercises	Sup.	60 min	75%	Health-related quality of life	-
Eggen et al., 2012 [25]	Norway	210	103	107	1	ND	16–20 w	Aerobic, strengthening, and pelvic floor exercises	Sup.	60 min	ND	Low-back and pelvic girdle pain	Pain, disability, and quality of life
Nascimento et al., 2011 [26]	Brazil	80	39	41	1–5	Low-Mod	17 w	Aerobic, stretching, strengthening, and relaxation exercises	Unsup. and Sup.	40 min	62.5%	Gestational weight gain	Arterial blood pressure, perinatal outcomes, and quality of life
O’Connor et al., 2018 [27]	USA	89	44	45	2	Low-Mod	12 w	Aerobic resistance and strengthening exercises	Unsup. and Sup.	17 min	78.4%	Quality of life and mood	Pain and physical function
Rodríguez-Blanque et al., 2020 [28]	Spain	129	65	64	3	Mod.	17 w	Aerobic, strengthening, endurance, stretching, and relaxation exercises	Sup.	60 min	80%	Sociodemographic, anthropometric, and perinatal outcomes. Quality of life, physical activity level, and intensity of exercise	-
Seneviratne et al., 2015 [29]	New Zealand	74	37	37	3–5	Mod.	15 w	Aerobic exercise (stationary cycling)	Unsup.	15–30 min	ND	Offspring birthweight	Prespecified maternal and perinatal outcomes (including quality of life)
Vázquez Lara et al., 2017 [30]	Spain	46	18	28	2	Mod.	6 w	Aerobic, pelvic floor, and relaxation exercises (in water)	Sup.	45 min	90%	Quality of life	-

Ref, Reference; IG, intervention group; CG, control group; Freq, weekly frequency; Intens, intensity; Mod, moderate; Durat, minutes of session duration; Time, weeks of intervention; Sup, supervised sessions; ND, not described; Sup, supervised; Unsup, unsupervised.

### 2.8. Statistical Analysis

Review Manager (RevMan version 5.4) software was used to perform two different meta-analyses related to self-perception of health status (i) focusing on the physical component or dimension and (ii) examining the mental or psychological dimension. As we included different QoL questionnaires, the overall confidence intervals (CIs) and standardized mean differences (SMDs) were calculated for these analyses using a random effects model. Effect sizes were calculated taking into account Hedges’ G, which was small (0.2), moderate (0.4), or large (0.8). Alpha error was set as 95%. 

For the meta-analyses, a relative weight, depending on the study group sample size, was assigned to each eligible article. I^2^ statistic was used to determine the presence of heterogeneity in each analysis, assessing the percentage of total variability attributable to study heterogeneity. Heterogeneity was considered low when I^2^ = 25%; moderate when I^2^ = 50%; and high when I^2^ = 75%. Since high heterogeneity was perceived in one meta-analysis, due to the limited number of eligible articles, subgroup analyses were not performed. 

## 3. Results

**Figure 1:**
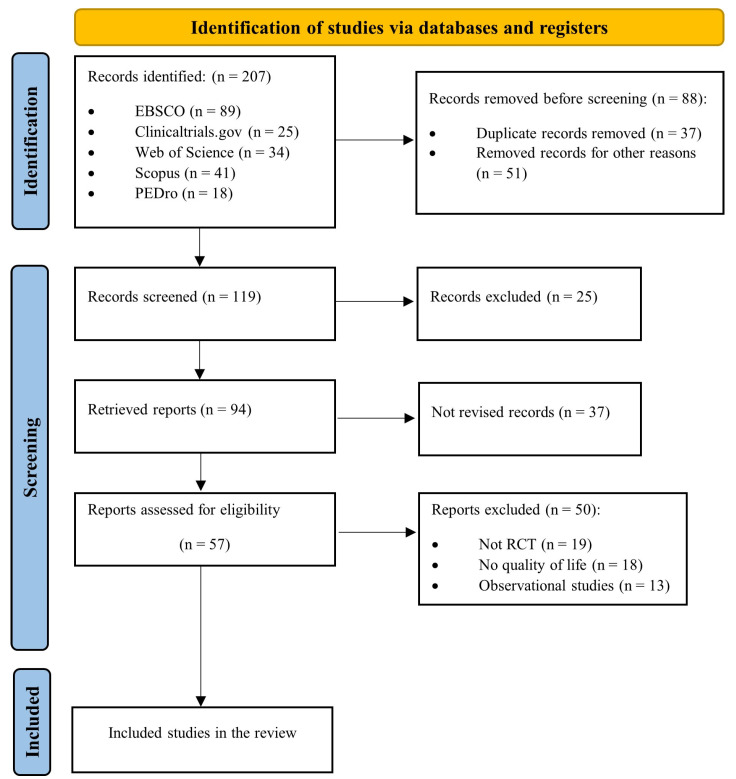
Flow diagram of included studies.

The PRISMA diagram below (Figure 1) illustrates the search results and study selection. A total of 207 abstracts were retrieved, with 88 being removed because of duplication or failure to meet the inclusion criteria. After additional screening, a total of 57 full-text articles were assessed for eligibility. Finally, data were extracted from seven [24,25,26,27,28,29,30] unique randomized clinical trials (n = 678 participants) that met our inclusion criteria and were incorporated in our two meta-analyses.

The included articles had physical activity intervention programs that lasted between 6 and 20 weeks, with weekly frequencies between one and five sessions comprising exercises of light and moderate intensity of 15 to 60 min of duration per session, including aerobic, strengthening, pelvic floor, stretching, and relaxation exercises. Six of seven interventions were supervised. The analyzed studies are described in Table 1.

### 3.1. Effects of Physical Activity on the Physical Component (PCS) of QoL Scores

**Figure 2:**
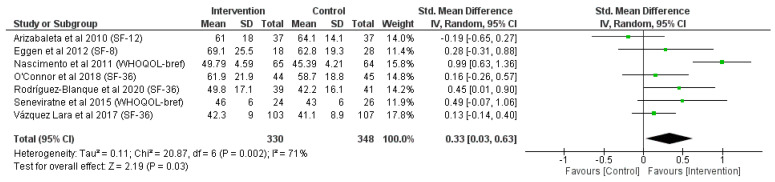
Meta-analysis of physical component of QoL questionnaire scores [24,25,26,27,28,29,30].

Seven articles included compatible questionnaire scores assessing the physical component/dimension of quality of life [24,25,26,27,28,29,30]. Overall, a significative improvement (z = 2.19; *p* = 0.03) in the physical component scores of the QoL questionnaires was shown for participants randomized to a physical activity intervention during pregnancy compared with the control (SMD = 0.33, 95% CI = 0.03, 0.63 I^2^ = 71%, P_heterogeneity_ = 0.002), as described in Figure 2.

### 3.2. Effects of Physical Activity on the Mental/Psychological Component (MCS) of QoL Scores

**Figure 3:**
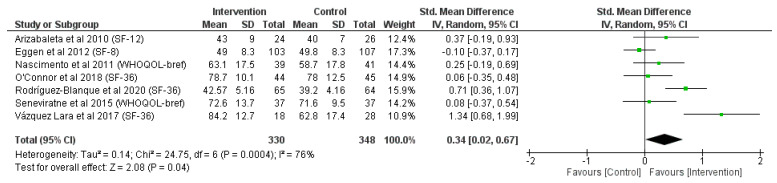
Meta-analysis of mental component of QoL questionnaire scores [24,25,26,27,28,29,30].

Overall, seven articles examined the mental component of QoL scores [24,25,26,27,28,29,30], showing a significative improvement (z = 2.08; *p* = 0.04) in women who performed a physical activity intervention during pregnancy compared with the control group, as represented in Figure 3 (SMD = 0.34, 95% CI = 0.02, 0.67, I^2^ = 76%, P_heterogeneity_ = 0.000).

### 3.3. Quality-of-Evidence and Risk-of-Bias Assessments

**Figure 4:**
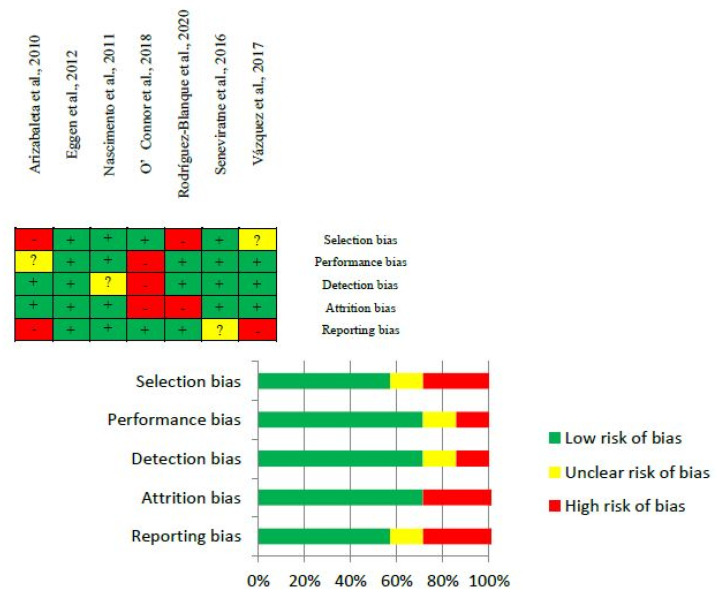
Risk-of-bias assessment of retrieved articles [24,25,26,27,28,29,30].

All the articles selected were randomized clinical trials. Due to the importance of the outcomes retrieved, the quality-of-evidence assessment resulted in high certainty and critical importance. The risk of bias varied between low, uncertain, and high in five different sources of bias (selection, performance, detection, attrition, and reporting), with more than 50% (in some cases, higher than 60%) having a low risk of bias among the sources, as is shown in Figure 4. Despite a high or unclear risk of bias being detected in some of the included studies, we did not deem the risk of bias to be a strong enough reason for discarding the selected articles.

## 4. Discussion

The aim of this systematic review and meta-analysis was to assess the effects of physical activity interventions during pregnancy on both the mental and physical components of self-perceived QoL; finally, seven articles (with four different questionnaires) were analyzed.

Our findings from the seven eligible RCTs suggest that there is “high”-quality evidence from physical activity interventions, indicating that prenatal PA could help pregnant women to improve their quality of life during their pregnancies with significantly better results in intervention group women than in control group participants. Specifically, our data showed improvements in both the physical and mental components of QoL. Although not assessing QoL per se, our results are consistent with previously published articles showing the mental health benefits of PA programs during pregnancy [17,18]. These results are extremely important due to women during pregnancy needing to have the best mental and physical health status in perfect balance to allow the fetus to growth and develop appropriately.

Prenatal PA is known to benefit health by reducing the odds of having macrosomia without increasing the odds of having a small newborn [14], while also improving maternal and fetal cardiac characteristics [31]. Then, PA could also positively influence QoL because mental health could improve thanks to its performance.

Interestingly, only one article [25] in the mental component of health status analysis and another [24] in the physical component analysis had better scores of health status in the control group than in the intervention group. Apparently, intervention programs lasting more than 15 weeks had moderate [25,28,29] and large [26] effect sizes compared with those whose interventions lasted less than 12 weeks [24,27,30] in Figure 2, suggesting that a larger physical activity intervention could better improve the physical component of health status. In addition, from Figure 3, it is possible to elucidate that the intervention participants of those articles involving unsupervised physical activity sessions [26,27,29] or carrying out just one weekly session [25] among their interventions had worse results in the mental component of self-perception of health status than those with supervised and more frequent weekly sessions in development articles [24,28,30]; it is logical to think that pregnant women that participate in a program with more weekly sessions and are under the supervision of a professional would have an improved mental health status. It is also possible that specific intervention characteristics played a role in the size of the response.

Heterogeneity was high in the physical component of QoL when high levels in the mental component meta-analysis were not reported. Although the mean age of the participants across the studies was nearly 30 years, there was one article [24] that had a mean age of 20 years, while another publication did not report the mean age [29,30]. The gestational age also varied between studies, where the included women were anywhere between 14 and 28 weeks at the study’s onset, with the last measure oscillating between 30 and 37 weeks of gestation. These age-related differences may be contributing to the high percentage of heterogeneity presented. While the included studies shared similar program characteristics, there were some dramatic differences in the program settings, thereby increasing heterogeneity. Also, the fact that four different questionnaires were used in the included studies (SF-8, SF-12, SF-36, and WHOQOL-BREF) could easily explain the high heterogeneity, and we were unable to perform subgroup analyses via a questionnaire due to the small number of eligible studies.

Our findings are aligned with other published systematic reviews, specifically a paper published by Lagadec et al. [6], whose aim was to address all the factors that could help pregnant women improve their quality of life. This systematic review confirmed that physical exercise is an indisputable requirement for a good perception of well-being. Another previously published review [32] showed that yoga interventions could significantly increase the perception of health status in pregnant women. Collectively, physical activity interventions is a simple and feasible method of improving QoL.

Considering the significance of maternal quality of life during pregnancy in enhancing the health of both the expectant mother and her future offspring, the outcomes of the current investigation substantiate the guidelines set forth by international bodies, advocating for a minimum of 150 min of moderate physical activity per week throughout pregnancy for women devoid of obstetric contraindications [33]. From an epidemiological standpoint, the present findings lend support to the concept of engaging in physical activity during gestation as a catalyst for comprehensive maternal health, encompassing all facets of her physiological, psychological, emotional, and social well-being.

Furthermore, the absence of moderate physical activity during pregnancy warrants recognition as a notable risk factor for significant complications and pathologies, resulting in a notable decline in the woman’s quality of life. This assertion finds validation in an extensive corpus of contemporary scientific literature and should be embraced by healthcare practitioners entrusted with the holistic care of pregnant women, as well as the pregnant population.

### Limitations and Strengths

One limitation in this study was the scarcity of articles published using PA as an intervention to improve quality of life during pregnancy. Also, the mental/psychological component of self-perception of health status analysis showed high heterogeneity, with an I^2^ of 76%. If we had been able to perform various subgroup analyses, then heterogeneity might have been attenuated, but the shortage of articles and the difficulty to assess a common factor included in all the articles to split them into subgroups made this option impossible. Additionally, QoL was assessed using various tools, necessitating the inclusion of all types in one analysis, thereby limiting our ability to perform subgroup analyses by tool. The risk of bias was generally low, except in some sources of bias that were had an unclear or high risk of bias. However, the quality of evidence and certainty of outcomes were categorized as high. Positively, seven articles were assessed representing six different countries and also five publications reported compliance to intervention above 62.5%. More physical activity intervention trials during pregnancy involving different self-perception of health status tools and retrieving other previous medical problems that could influence QoL are needed for a more comprehensive overview that could address the impact of exercise type, intensity, frequency, and duration.

## 5. Conclusions

Physical activity interventions supervised by a professional could have an important effect on improving both mental and physical self-perceived health status of pregnant women without contraindications.

## Data Availability

Not applicable.

## References

[1] Lebel C., MacKinnon A., Bagshawe M., Tomfohr-Madsen L., Giesbrecht G. (2020). Elevated depression and anxiety symptoms among pregnant individuals during the COVID-19 pandemic. J. Affect. Disord..

[2] Ding X., Liang M., Wang H., Song Q., Guo X., Su W., Li N., Liu H., Ma S., Zhou X. (2023). Prenatal stressful life events increase the prevalence of postpartum depression: Evidence from prospective cohort studies. J. Psychiatr. Res..

[3] World Health Organization (2012). WHOQOL: Measuring Quality of Life. https://www.who.int/tools/whoqol.

[4] Wu H., Sun W., Chen H., Wu Y., Ding W., Liang S., Huang X., Chen H., Zeng Q., Li Z. (2021). Health-related quality of life in different trimesters during pregnancy. Health Qual. Life Outcomes.

[5] Bjelica A., Cetkovic N., Trninic-Pjevic A., Mladenovic-Segedi L. (2018). The phenomenon of pregnancy—A psychological view. Ginekol. Pol..

[6] Lagadec N., Steinecker M., Kapassi A., Magnier A.M., Chastang J., Robert S., Gaouaou N., Ibanez G. (2018). Factors influencing the quality of life of pregnant women: A systematic review. BMC Pregnancy Childbirth.

[7] Sun W., Huang X., Wu H., Zhang C.S.P., Yin Z., Fan Q., Wang H., Jayavanth P., Akinwinmi B., Wu Y. (2021). Maternal tobacco exposure and health-related quality of life during pregnancy: A national-based study of pregnant women in China. Health Qual. Life Outcomes.

[8] Aydin Ü., Eser F., Garip Y. (2015). Impact of functional status on the quality of life of pregnant women with lumbopelvic pain. Istanb. Med. J..

[9] Amador N., Juárez J.M., Guízar J.M., Linares B. (2008). Quality of life in obese pregnant women: A longitudinal study. Am. J. Obstet. Gynecol..

[10] Bo H.X., Yang Y., Zhang D.Y., Zhang M., Wang P.H., Liu X.H., Ge L.N., Lin W.X., Xu Y., Zhang Y.L. (2021). The prevalence of depression and its association with quality of life among pregnant and postnatal women in China: A Multicenter Study. Front. Psychiatr..

[11] Berber M.A., Satılmış İ.G. (2020). Characteristics of low back pain in pregnancy, risk factors, and its effects on quality of life. Pain Manag. Nurs..

[12] Niles A., O’Donovan A. (2019). Comparing anxiety and depression to obesity and smoking as predictors of major medical illnesses and somatic symptoms. Health Psychol..

[13] Ribeiro M.M., Andrade A., Nunes I. (2021). Physical exercise in pregnancy: Benefits, risks and prescription. J. Perinat. Med..

[14] Wiebe H.W., Boule N.G., Chari R., Davenport M.H. (2015). The effect of supervised prenatal exercise on fetal growth: A meta-analysis. Obstet. Gynecol..

[15] Nagpal T.S., Bhattacharjee J., da Silva D.F., Souza S.C., Mohammad S., Puranda J.L., Abu-Dieh A., Cook J., Adamo K.B. (2021). Physical activity may be an adjuvant treatment option for substance use disorders during pregnancy: A scoping review. Birth Defects Res..

[16] Davenport M.H., Ruchat S.-M., Sobierajski F., Poitras V.J., Gray C.E., Yoo C., Skow R.J., Garcia A.J., Barrowman N., Meah V.L. (2019). Impact of prenatal exercise on maternal harms, labour and delivery outcomes: A systematic review and meta-analysis. Br. J. Sports Med..

[17] Sánchez-Polán M., Franco E., Silva-José C., Gil-Ares J., Pérez-Tejero J., Barakat R., Refoyo I. (2021). Exercise during pregnancy and prenatal depression: A systematic review and meta-analysis. Front. Physiol..

[18] Davenport M.H., McCurdy A.P., Mottola M.F., Skow R.J., Meah V.L., Poitras V.J., Garcia A.J., Gray C.E., Barrowman N., Riske L. (2018). Impact of prenatal exercise on both prenatal and postnatal anxiety and depressive symptoms: A systematic review and meta-analysis. Br. J. Sports Med..

[19] ACOG Committee on Obstetric Practice (2002). “ACOG Committee Opinion. Number 267, January 2002.” Exercise during pregnancy and the postpartum period. Obstet. Gynecol..

[20] ACOG Committee on Obstetric Practice (2015). “ACOG Committee Opinion. Number 650, December 2015.” Physical Activity and Exer-cise During Pregnancy and the Postpartum Period. Obstet. Gynecol..

[21] GRADEpro Guideline Development Tool (2022). McMaster University and Evidence Prime. gradepro.org.

[22] Guyatt G.H., Oxman A., Vist G., Kunz R., Falck-Ytter Y., Alonso-Coello P., Schünemann H.J. (2008). GRADE an Emerging Consensus on Rating Quality of Evidence and Strength of Recommendations. BMJ.

[23] Higgins J.P., Savović J., Page M.J., Elbers R.G., Sterne J.A. (2019). Assessing risk of bias in a randomized trial. Cochrane Handbook for Systematic Reviews of Interventions.

[24] Arizabaleta A.V.M., Buitrago L.O., de Plata A.C.A., Escudero M.M., Ramírez-Vélez R. (2010). Aerobic exercise during pregnancy improves health-related quality of life: A randomised trial. J. Physiother..

[25] Eggen M.H., Stuge B., Mowinckel P., Jensen K.S., Hagen K.B. (2012). Can supervised group exercises including ergonomic advice reduce the prevalence and severity of low back pain and pelvic girdle pain in pregnancy? A randomized controlled trial. Phys. Ther..

[26] Nascimento S.L., Surita F.G., Parpinelli M.A., Siani S., Pinto e Silva J.L. (2011). The effect of an antenatal physical exercise programme on maternal/perinatal outcomes and quality of life in overweight and obese pregnant women: A randomised clinical trial. BJOG Int. J. Obstet. Gynaecol..

[27] O’Connor P.J., Poudevigne M.S., Johnson K.E., De Araujo J.B., Ward-Ritacco C.L. (2018). Effects of resistance training on fatigue-related domains of quality of life and mood during pregnancy: A randomized trial in pregnant women with back pain. Psychosom. Med..

[28] Rodríguez-Blanque R., Aguilar-Cordero M.J., Marín-Jiménez A.E., Menor-Rodríguez M.J., Montiel-Troya M., Sánchez-García J.C. (2020). Water exercise and quality of life in pregnancy: A randomised clinical trial. Int. J. Environ. Res. Public Health.

[29] Seneviratne S.N., Jiang Y., Derraik J.G.B., McCowan L.M.E., Parry G.K., Biggs J.B., Craigie S., Gusso S., Peres G., Rodrigues R.O. (2016). Effects of antenatal exercise in overweight and obese pregnant women on maternal and perinatal outcomes: A randomised controlled trial. BJOG Int. J. Obstet. Gynaecol..

[30] Vázquez Lara J.M., Rodríguez Díaz L., Ramírez Rodrigo J., Villaverde Gutiérrez C., Torres Luque G., Gómez-Salgado J. (2017). Calidad de vida relacionada con la salud en una población de gestantes sanas tras un programa de actividad física en el medio acuático (PAFMAE). Rev. Esp. Salud Pública.

[31] Moyer C., May L. (2014). Influence of exercise mode on maternal and fetal health outcomes (886.3). FASEB J..

[32] Corrigan L., Moran P., McGrath N., Eustace-Cook J., Daly D. (2022). The characteristics and effectiveness of pregnancy yoga interventions: A systematic review and meta-analysis. BMC Pregnancy Childbirth.

[33] Bull F.C., Al-Ansari S.S., Biddle S., Borodulin K., Buman M.P., Cardon G., Carty C., Chaput J.P., Chastin S., Chou R. (2020). World Health Organization 2020 guidelines on physical activity and sedentary behaviour. Br. J. Sports Med..

